# PRL-3 disrupts epithelial architecture by altering the post-mitotic midbody position

**DOI:** 10.1242/jcs.190215

**Published:** 2016-11-01

**Authors:** Pablo Luján, Giulia Varsano, Teresa Rubio, Marco L. Hennrich, Timo Sachsenheimer, Manuel Gálvez-Santisteban, Fernando Martín-Belmonte, Anne-Claude Gavin, Britta Brügger, Maja Köhn

**Affiliations:** 1 European Molecular Biology Laboratory, Genome Biology Unit, Heidelberg 69117, Germany; 2 European Molecular Biology Laboratory, Structural and Computational Biology Unit, Heidelberg 69117, Germany; 3 Heidelberg University Biochemistry Center, University of Heidelberg, Heidelberg 69120, Germany; 4 Department of Development and Differentiation, Centro de Biología Molecular Severo Ochoa, Consejo Superior de Investigaciones Científicas (CSIC), Madrid 28049, Spain

**Keywords:** PRL-3, PTP4A3, Midbody, Cell polarity, Epithelia, Cancer

## Abstract

Disruption of epithelial architecture is a fundamental event during epithelial tumorigenesis. We show that the expression of the cancer-promoting phosphatase PRL-3 (PTP4A3), which is overexpressed in several epithelial cancers, in polarized epithelial MDCK and Caco2 cells leads to invasion and the formation of multiple ectopic, fully polarized lumens in cysts. Both processes disrupt epithelial architecture and are hallmarks of cancer. The pathological relevance of these findings is supported by the knockdown of endogenous PRL-3 in MCF-7 breast cancer cells grown in three-dimensional branched structures, showing the rescue from multiple-lumen- to single-lumen-containing branch ends. Mechanistically, it has been previously shown that ectopic lumens can arise from midbodies that have been mislocalized through the loss of mitotic spindle orientation or through the loss of asymmetric abscission. Here, we show that PRL-3 triggers ectopic lumen formation through midbody mispositioning without altering the spindle orientation or asymmetric abscission, instead, PRL-3 accelerates cytokinesis, suggesting that this process is an alternative new mechanism for ectopic lumen formation in MDCK cysts. The disruption of epithelial architecture by PRL-3 revealed here is a newly recognized mechanism for PRL-3-promoted cancer progression.

## Abstract (precis)

**Summary:** The cancer-promoting phosphatase PRL-3 triggers ectopic lumen formation through midbody mispositioning by accelerating cytokinesis, suggesting a new oncogenic mechanism.

## INTRODUCTION

Defects in polarized epithelial tissue architecture are the source of more than 80% of human cancers, and deregulation or loss of polarity in epithelial cells is considered fundamental during tumor progression (Datta et al., 2011; McCaffrey and Macara, 2011; St Johnston and Ahringer, 2010). Although the phosphatase of regenerating liver-3 (PRL-3; also known as PTP4A3) is not found in most adult tissues, its overexpression is known to promote epithelial tumor progression, leading to poor outcome (Guzińska-Ustymowicz and Pryczynicz, 2011; Rios et al., 2013). Moreover, PRL-3 is an emerging prognostic marker for the prediction of human colorectal cancer progression (Guzińska-Ustymowicz and Pryczynicz, 2011). Nevertheless, PRL-3 has not yet been investigated in the context of epithelial architecture and polarity (Rios et al., 2013). PRL-3 belongs to the family of the PRL proteins within the dual specificity phosphatase (DSP) family. The PRL family comprises three members, PRL-1, PRL-2 and PRL-3, and all have been implicated in promoting cancer progression (Rios et al., 2013). PRL-3 localizes to membrane compartments, mainly the plasma membrane. Protein and non-protein substrates have been suggested for PRL-3, but none has yet been established as a substrate that would explain the PRL-3-dependent phenotypes (Rios et al., 2013). PRL-3 has been shown to promote proliferation and invasion in two-dimensional (2D) cell cultures (Al-Aidaroos and Zeng, 2010; Rios et al., 2013).

Epithelial polarization and *de novo* lumen formation have been extensively studied in 3D-cultured Madin-Darby canine kidney (MDCK; non-cancerous), Caucasian colon adenocarcinoma (Caco-2) and MCF-7 (Caucasian breast adenocarcinoma) cells (Apodaca, 2010; Bryant et al., 2010, 2014; do Amaral et al., 2011; Gálvez-Santisteban et al., 2012; Jaffe et al., 2008; Lee et al., 2007; Rodríguez-Fraticelli et al., 2015; Schlüter and Margolis, 2009). In contact with extracellular matrix, MDCK and Caco-2 cells form polarized spherical cysts with the apical membrane facing a single central lumen. Similarly, MCF-7 cells form branched tubular structures where lumens are opened in the branch ends, surrounded by polarized cells with the apical membrane facing the lumen. *De novo* lumen formation in MDCK cysts starts during the first mitosis and requires polarized exocytosis of vesicles containing apical markers along the mitotic spindle to the site of abscission (Dionne et al., 2015; Li et al., 2014; Wang et al., 2014). Then, the apical membrane initiation site (AMIS) (Apodaca et al., 2012; Bryant et al., 2010) is formed around the midbody (Apodaca et al., 2012; Li et al., 2014; Schlüter et al., 2009; Wang et al., 2014), a structure created within the intercellular bridge during cytokinesis (D'Avino and Capalbo, 2016; Overeem et al., 2015; Schlüter and Margolis, 2009; Steigemann and Gerlich, 2009), where the apical membrane will finally be positioned (Jaffe et al., 2008). In subsequent cell divisions, the central single lumen is maintained through planar orientation of the mitotic spindle during metaphase, generating a radial cleavage furrow. This furrow ingresses asymmetrically to locate the midbody at the apical membrane at the end of the abscission (Bryant et al., 2010; Jaffe et al., 2008; Li et al., 2014; Overeem et al., 2015; Schlüter et al., 2009). Loss of spindle orientation or of asymmetric abscission results in mislocalized midbodies, leading to ectopic lumen formation and thus disruption of epithelial architecture (Jaffe et al., 2008; Schlüter et al., 2009). Although loss of spindle orientation has been extensively studied (Jaffe et al., 2008; Overeem et al., 2015), spindle orientation-independent mislocalization of midbodies remains as a theoretical scenario (Jaffe et al., 2008; Overeem et al., 2015; Schlüter et al., 2009), leaving room for alternative mechanisms.

Here, we show that both the overexpression of PRL-3 in MDCK and Caco-2 cysts and high levels of PRL-3 in MCF-7 structures disrupt epithelial morphogenesis. PRL-3 alters the position of post-mitotic midbodies (midbody remnants) without altering spindle orientation or asymmetric cleavage furrow ingression, suggesting that an alternative mechanism can occur. Our studies reveal that PRL-3 enhances the pace of cytokinesis, leading to the hypothesis that PRL-3 prevents the apical localization of the midbody through early abscission, resulting in a new pathway for the disruption of epithelial architecture. Furthermore, we show that in MDCK cysts, the midbody is retained in the apical membrane long after cell division, supporting a role for the midbody in polarity maintenance in these cells besides that in *de novo* lumen formation, as has been previously reported for this cell system (Jaffe et al., 2008).

## RESULTS

### PRL-3 overexpression affects lumen formation in epithelial cells

**Fig. 1.**
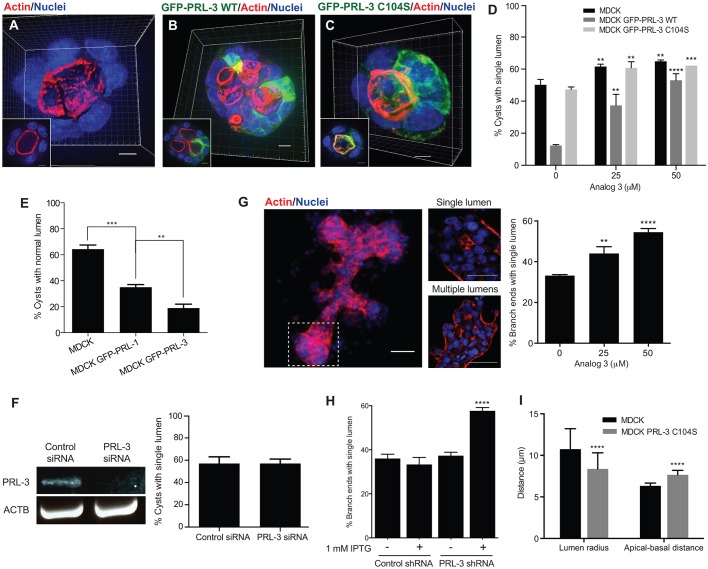
**PRL-3 expression in epithelial cells leads to the formation of aberrant multiple-lumen-containing cysts.** (A–C) 3D reconstruction of parental (A) MDCK cysts and of MDCK cysts overexpressing GFP–PRL-3-WT (B) or GFP–PRL-3-C104S (C) (green) at 72 h. Scale bars: 10 μm. Representative 2D plane from the selected cysts is shown in the bottom-left corner. Blue, nuclei; red, actin. Scale bars: 5 µm. (D) Percentage of cysts with a single lumen in 72-h MDCK cysts or cysts that stably expressed GFP–PRL-3-WT or GFP–PRL-3-C104S. Cells were treated with analog 3 or DMSO (control, 0 µM analog 3). *n*=300. (E) Percentage of cysts with a single lumen in 72-h MDCK cysts that transiently expressed GFP–PRL-1-WT or GFP–PRL-3-WT. *n*=300. (F) Proportion of 48-h single-lumen MDCK cysts that had been electroporated with control siRNA or siRNA against PRL-3. PRL-3 mRNA was assayed by performing one-step RT-PCR using mRNA encoding β-actin as a loading control, *n*=300. (G,H) 3D reconstruction of an MCF-7 branched tubular structure after 15 days of growth. Dashed square highlights a branch end that presents a single lumen (middle panel, top) or multiple lumens (middle panel, bottom). Graph represents the proportion of branch ends that exhibited a single lumen. Cells were treated with analog 3 or DMSO (control, 0 µM analog 3) (G) or with 1 mM IPTG for MCF-7 cells that stably expressed IPTG-inducible control shRNA or shRNA against PRL-3 (H). *n*=300. Scale bars: 100 µm (left), 50 µm (middle column). (I) Lumen radius, and distance between apical and basal membranes measured in parental and GFP–PRL-3-C104S MDCK cysts. *n*=90. Values in D–I represent mean±s.d. of three biological replicates. **P≤0.01, ***P≤0.001 and ****P≤0.0001 (D,I, ordinary two-tailed two-way ANOVA with Tukey's multiple comparison test; E,G,H, ordinary two-tailed one-way ANOVA with Tukey's multiple comparison test; F, *t*-test compared to control siRNA).

Over the past few years, several reports have shown a correlation between high pathological PRL-3 expression and tumor progression of epithelial cancers (reviewed in Al-Aidaroos and Zeng, 2010; Guzińska-Ustymowicz and Pryczynicz, 2011; Rios et al., 2013), but the effect of aberrant PRL-3 expression on cellular polarity has not yet been examined. To elucidate whether PRL-3 activity induces a cancer-relevant phenotype in polarized epithelial cells, we stably overexpressed the wild-type (WT) version of PRL-3 fused to GFP (GFP–PRL-3-WT) in MDCK and Caco-2 cells. As previously reported, MDCK and Caco-2 cells form spherical cysts in Matrigel that are characterized by a central hollow lumen surrounded by a single layer of polarized cells (Apodaca et al., 2012; Debnath and Brugge, 2005; Li et al., 2013). In these cysts (Fig. 1A,D; Fig. S1A,D), actin was enriched below the apical membrane facing the central lumen (Horikoshi et al., 2009). By contrast, PRL-3-overexpressing cysts were frequently abnormal, with multiple lumens that were still positive for actin (Fig. 1B,D; Fig. S1B,D). This defect depended on PRL-3 catalytic activity because MDCK and Caco-2 cysts that stably expressed the catalytically inactive mutant of PRL-3 (GFP–PRL-3-C104S) showed a phenotype that was comparable to parent-line (parental) cysts (Fig. 1C,D; Fig. S1C,D). To exclude that this finding is due to different expression levels, we compared the expression levels of PRL-3 WT and C104S in the stable cell lines (Fig. S1E). Because PRL-3 protein levels in cells are tightly regulated (Choi et al., 2011; Xing et al., 2016), which leads to heterogeneous PRL-3 expression in the MDCK stable cell lines (Fig. 1B,C), we used flow cytometry to study the expression distribution of PRL-3 in these cell lines (Fig. S1E). We observed that the proportion of MDCK cells expressing the highest levels of GFP–PRL-3-C104S was increased compared to the proportion of cells expressing the highest levels of PRL-3-WT, suggesting that PRL-3 protein level regulation might depend on PRL-3 activity. This finding also demonstrates that the PRL-3-C104S phenotype is not due to a general decrease of protein expression compared to that of WT.

In agreement with the single lumen phenotype observed for PRL-3-C104S-overexpressing cysts, we observed a concentration-dependent rescue towards the normal phenotype (Fig. 1D; Fig. S1D) when PRL-3-WT-overexpressing cysts were treated with the selective PRL inhibitor analog 3 (Hoeger et al., 2014). The parental and the GFP–PRL-3-C104S MDCK stable cell lines also showed a slightly increased number of single-lumen-containing cysts when treated with analog 3 (Fig. 1D), but not the corresponding Caco-2 cells (Fig. S1D). This could be due to the endogenous presence of PRL proteins in MDCK cells because analog 3 inhibits all PRLs (Hoeger et al., 2014). Endogenous dog PRL-3 protein could neither be detected by western blot analysis in MDCK cells using different antibodies (see Materials and Methods) nor by deep mass-spectrometry (MS) analysis, which is used to detect low-expressing proteins; by contrast, human PRL-3 in the cells overexpressing human GFP–PRL-3-WT and GFP–PRL-3-C104S were identified. However, peptides corresponding to endogenous PRL-1 and PRL-2 were found, suggesting that the observed slight increase could be due to endogenous PRL-1 and/or PRL-2. To this end, PRL-1 was transiently overexpressed in MDCK cysts. Like PRL-3, it also caused the formation of multiple lumens, although to a lesser extent (Fig. 1E; Fig. S1F–H), supporting the above hypothesis and indicating that endogenous PRLs are able to exert the observed effect on lumen formation. Nevertheless, to address whether endogenous PRL-3 could have an effect on lumen formation at low protein levels that are undetectable by MS and antibody-mediate analyses, we knocked down PRL-3 by using RNA interference (Fig. S1I) in MDCK and Caco-2 cysts. No change was observed in cyst formation compared to that in parental cysts (Fig. 1F; Fig. S1J), supporting that PRL-3 protein is not present or expressed at very low protein levels in these cell lines. In agreement with this, PRL-3 mRNA and protein levels have been reported previously to not always correlate because translational and post-translational regulation can tightly control protein levels (Choi et al., 2011; Wang et al., 2010; Xing et al., 2016).

Given that PRL-3 protein was not detected in MDCK and Caco-2 cells, it was not possible to study a potential endogenous role of PRL-3 using these cell systems. Thus, we used MCF-7 breast cancer cells, the only cell line where detectable levels of PRL-3 protein have been reported (Geiger et al., 2012; Wang et al., 2010) and that can be cultured to grow in three dimensions and to form lumen-containing structures (do Amaral et al., 2011; Lee et al., 2007). In the branch ends of these structures, single or multiple lumens opened, and these lumens were positive for actin (Fig. 1G). When the cells were treated with the PRL inhibitor analog 3, the proportion of single-lumen-containing branch ends increased in a concentration-dependent manner (Fig. 1G). This was also the case when PRL-3 was stably knocked down (Fig. 1H; Fig. S1K). This result corroborates the phenotype observed with PRL-3 overexpression and suggests a pathophysiological role of PRL-3 in disrupting epithelial architecture.

PRL-3-mediated cell proliferation and invasion, also hallmarks of cancer cells, have never been studied in polarized cells. To test for an invasive phenotype, MDCK parental and GFP–PRL-3-overexpressing cysts were grown in Matrigel–collagen-I (1:1) (Kwon et al., 2011). The MDCK GFP–PRL-3-WT cysts showed a tubular invasive phenotype compared to that of parental and GFP–PRL-3-C104S MDCK cysts (Fig. S1L–O). Regarding proliferation, the size of MDCK GFP–PRL-3-WT cysts, measured at 72 h, showed no difference in comparison to the size of parental and GFP–PRL-3-C104S MDCK cysts (Fig. S1P), suggesting that PRL-3 does not induce proliferation in this system up to this stage. Interestingly, GFP–PRL-3-C104S MDCK cysts developed smaller lumens than the parental cysts, caused by an increased length of the apical–basal axis of the cells (Fig. 1I), which could be an intermediate phenotype between the normal phenotype of MDCK cysts and the multi-lumen phenotype of MDCK GFP–PRL-3-WT cysts. This could be induced by a substrate-trapping effect, as GFP–PRL-3-C104S can potentially still bind to but not process its substrates.

Taken together, these results show that aberrant PRL-3 expression, mimicking the pathological expression in epithelial cancers, has a detrimental effect on epithelial architecture and increases cell invasion. Because high levels of PRL-3 also induce multiple lumens in MCF-7 breast cancer 3D cell structures, low abundance or absence of PRL-3 might be needed in healthy epithelial cells to ensure correct tissue formation. In the following, we focused on studying the disruption of epithelial architecture in MDCK cysts caused by PRL-3 activity, as this is a newly discovered phenotype.

### PRL-3-induced fully polarized ectopic lumens arise from PRL-3-enriched incorrectly localized AMISs

**Fig. 2.**
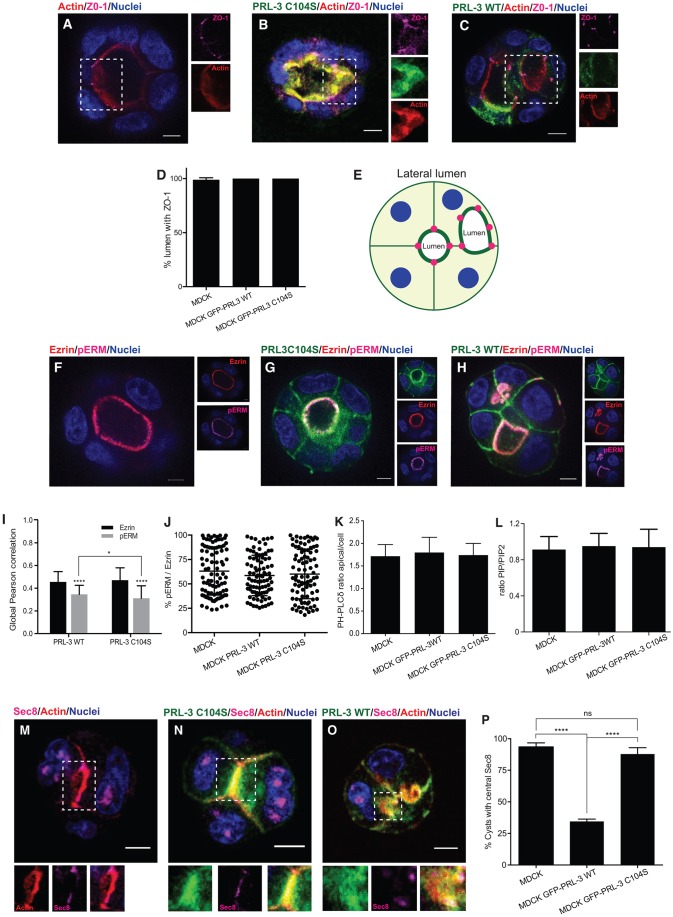
**PRL-3-overexpressing cysts show fully specified ectopic lumens that arise from PRL-3-enriched incorrectly localized AMISs.** (A–D) Localization of ZO-1 (magenta) in a 72-h MDCK cyst (A) and cysts expressing GFP–PRL-3-C104S (B) or GFP–PRL-3-WT (C) (both proteins in green). Blue, nuclei; red, actin. Right: separated fluorescence channels of the indicated regions showing the indicated staining. Scale bars: 10 µm. (D) The proportion of cysts with ZO-1 staining in the nascent lumen. *n*=90. See also Movie 1. (E) Schematic representation of MDCK cells overexpressing PRL-3-WT showing an asymmetric invasive ectopic lumen flanked with ZO-1 (magenta dots). (F–H) Experiments as described in A–C, except cysts were stained with antibodies against ezrin (red) and threonine-phosphorylated ERM (ezrin-radixin-moesin) proteins (pERM) (magenta). Right: separate fluorescent channels showing the indicated staining. Scale bars: 10 µm. (I) Global Pearson correlation was measured to study colocalization between PRL-3-WT or PRL-3-C104S and ezrin or pERM. *n*=90. (J) Ezrin dephosphorylation was determined by calculating the fluorescence intensity ratio between pERM and ezrin in parental, GFP–PRL-3-WT and GFP–PRL-3-C104S MDCK cysts. *n*=90. (K) PI(4,5)P_2_ dephosphorylation in apical membranes was determined by calculating the ratio between apical and the combined cytosolic and basal fluorescence intensity of GFP–PH-PLCδ in parental, GFP–PRL-3-WT and GFP–PRL-3-C104S MDCK cysts. *n*=90. (L) Quantitative lipid MS analysis of MDCK cysts or cysts that stably expressed GFP–PRL-3-WT or GFP–PRL-3-C104S analyzed 5 days after seeding. The ratio between the total phosphatidylinositol phosphates PIP and PIP_2_ was quantified. (M–P) Localization of Sec8 (magenta) in 48-h MDCK cysts with nascent lumen. Parental cysts (M) and cysts that stably expressed PRL-3-C104S (N) or PRL-3-WT (O) are shown. Blue, nuclei; red, actin. Bottom: focus on the indicated regions showing individual channels of the indicated GFP–PRL-3 construct or actin (left), Sec8 (middle) and the merged image (right). (P) The proportion of cysts with central Sec8 staining. *n*=90. Scale bars: 10 µm. Values in D,I,K,L,P represent the mean±s.d. of three biological replicates. ns, not significant; ****P≤0.0001 (D,J,K,L,P, ordinary two-tailed one-way ANOVA with Tukey's multiple comparison test; I, ordinary two-tailed two-way ANOVA with Tukey's multiple comparison test).

MDCK cells establish apical–basal polarity at a very early stage of cystogenesis, when the membranes that face the center of the cyst acquire a clear apical identity and well-defined tight junctions are formed right after the first cell division (Apodaca, 2010; Apodaca et al., 2012; Bryant et al., 2010). To further characterize PRL-3-induced ectopic lumens, we investigated the localization of tight junctions in PRL-3-WT cysts. The tight junction marker ZO-1 (also known as TJP1) localized to the apical sites of cell–cell contact that were adjacent to the central lumen in parental and GFP–PRL-3-C104S MDCK cysts (Fig. 2A,B,D; Fig. S2A,B), as previously reported for the parental cysts (Bryant et al., 2010; Martin-Belmonte et al., 2007). PRL-3-induced ectopic lumens were still flanked by tight junctions (Fig. 2C–E; Fig. S2C). 3D analysis of 660 cysts showed that these ectopic lumens are shared by two or more cells, thus they seem to arise between lateral membranes (Movie 1). They can be symmetric between both cells or invasive towards one of the cells (Fig. 2C,E; Fig. S2C, Movie 1). In agreement with the intact tight junctions observed in the PRL-3-overexpressing cysts, *de novo* tight junction formation was undisturbed by PRL-3 in a *de novo* tight junction formation assay (Fig. S2D).

Two of the suggested PRL-3 substrates, phosphatidylinositol 4,5 bisphosphate [PI(4,5)P_2_] (McParland et al., 2011) and ezrin (Forte et al., 2008), are particularly interesting with respect to their potential involvement in the mechanism by which PRL-3 could cause this phenotype because they are known determinants of cellular polarity (Martin-Belmonte et al., 2007), marking the apical membrane. In MDCK parental and PRL-3-overexpressing cysts, ezrin was present in the apical membrane and partially colocalized with overexpressed GFP–PRL-3-WT and GFP–PRL-3-C104S (Fig. 2F–I; Fig. S2E). Compared to ezrin, phosphorylated ezrin colocalized to a lesser extent with both PRL-3-WT and with PRL-3-C104S (Fig. 2I), suggesting that this difference was not due to dephosphorylation. Consistent with this, there was no difference in the ratio between the fluorescence intensity of phosphorylated ezrin and ezrin in parental, GFP–PRL-3-WT- and GFP-PRL-3-C104S-expressing cysts (Fig. 2J). Similarly, PI(4,5)P_2_ was present at the apical membrane in parental and mKate–PRL-3-overexpressing cysts and colocalized with mKate–PRL-3 (Fig. S2F–H). However, no obvious differences in the fluorescence intensity of the PI(4,5)P_2_ sensor GFP–PH-PLCδ at the apical membrane was detected (Fig. 2K). The overall phosphatidylinositol bisphosphate (PIP_2_) content in MDCK cysts was also measured by performing quantitative MS, with which one cannot distinguish between the three PIP_2_ species. In comparison with controls, no difference was detected when GFP–PRL-3-WT was overexpressed (Fig. 2L; Fig. S2I). Interestingly, however, when overexpressing the phosphatase PRL-1, which cannot dephosphorylate PI(4,5)P_2_ (Rios et al., 2013; Sun et al., 2007), we observed a similar but weaker phenotype compared to that upon PRL-3 overexpression (Fig. 1E), with similar expression levels of PRL-1 and PRL-3 (Fig. S1F–H). This difference could thus be due to the different activities toward PI(4,5)P_2_, but other effects cannot be excluded.

Because the ectopic lumens appeared to be fully specified, and tight junction formation was undisturbed, we hypothesized that PRL-3 catalytic activity might rather lead to a failure in lumen positioning. AMIS specification is one of the earliest steps in lumenogenesis and precedes the formation of a tight-junction-delimited lumen (Apodaca, 2010; Bryant et al., 2010). As expected, in parental and PRL-3-C104S MDCK cysts, the AMIS marker Sec8 was present at cell–cell contacts at the center of the cysts – the site at which the lumen opened (Fig. 2M,N,P). In PRL-3-WT-overexpressing cysts, Sec8 localized to PRL-3 patches, suggesting that the AMIS is properly formed before lumen opening (Fig. S2J). However, the AMIS was mislocalized from the center of the cyst to the lateral membrane (Fig. 2O,P).

Taken together, these results indicate that PRL-3 catalytic activity leads to a failure in the specification of the lumen position rather than to a disruption in overall cell polarity, giving rise to fully polarized lumens in ectopic positions. Therefore, PRL-3 appears to interfere at an early step in lumenogenesis.

### PRL-3 affects lumen positioning by altering the post-mitotic midbody localization

It has been described previously that the AMIS is formed around the midbody at the site of and during cytokinesis (Li et al., 2014). It has, furthermore, been reported that the midbody positions the apical membrane during and right after cytokinesis (Jaffe et al., 2008; Overeem et al., 2015; Schlüter et al., 2009), but its fate afterwards remains unstudied in MDCK and Caco-2 cysts. Nevertheless, it has been stated that post-mitotic midbodies can participate in functions unrelated to cytokinesis in other cell types (Chen et al., 2013; Kuo et al., 2011; Morais-de-Sá and Sunkel, 2013; Pollarolo et al., 2011).

**Fig. 3.**
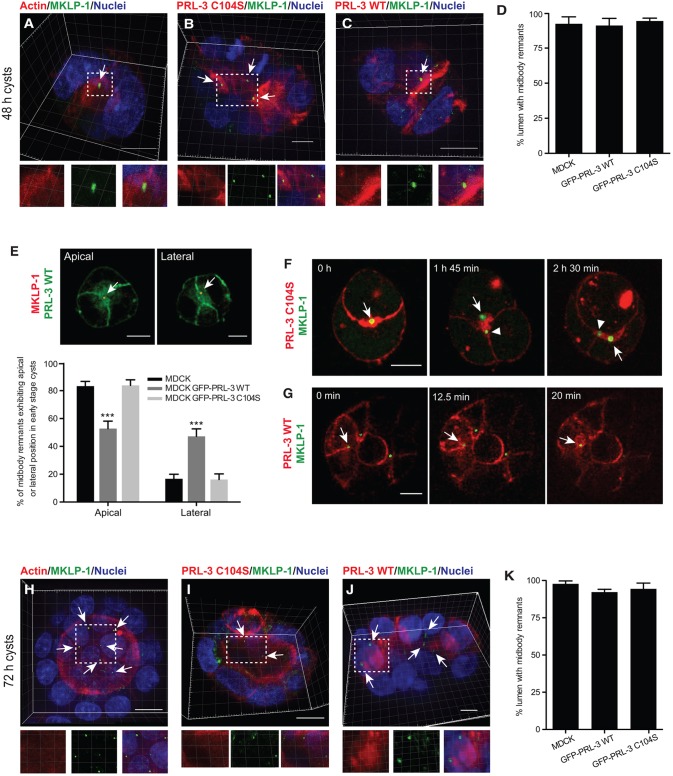
**PRL-3 activity alters the position of the post-mitotic midbody.** (A–D) 3D reconstruction images of 48-h parent cysts (A), of MDCK cysts overexpressing GFP–PRL-3-C104S (B) or GFP–PRL-3-WT (C) (both in red) and stained for MKLP1 (green) to visualize midbody remnants. Nuclei, blue; actin, red in A. Bottom: individual channels (left, middle) and merged images (right) of the indicated regions. Arrows indicate midbody remnants. (D) The proportion of cysts presenting midbodies in the nascent lumen. *n*=90. Scale bars: 10 µm. (E) Midbody remnants (MKLP1, red) in 48-h cysts expressing GFP–PRL-3 WT (green). Arrows indicate midbody remnants with central (apical, left) or lateral (right) localization. The proportion of midbody remnants showing central (apical) or lateral localization in MDCK cysts and cysts that stably expressed GFP–PRL-3-WT and GFP–PRL-3-C104S were analyzed at 48 h after seeding. *n*=90. (F,G) Selected time points during live-cell imaging of 45-h cysts that transiently co-expressed either mKATE-PRL-3-C104S (H) or mKATE-PRL-3-WT (I) and YFP–MKLP1. The arrow in F highlights the first midbody remnant; arrowhead highlights the midbody remnant coming from the second cytokinesis event. Arrow in G indicates a midbody remnant that gets retained at the lateral plasma membrane with subsequent ectopic lumen formation. Representative results of three biological replicates are shown. See also Movies 2 and 3. Scale bars: 10 µm. (H–K) Experiments were performed as described in A–D, except cysts were analyzed at 72 h after seeding. Arrows indicate midbody remnants. Bottom: individual channels (left, middle) and merged images (right) of the indicated regions. Data are mean±s.d. *n*=90. ***P≤0.001 (D,K, ordinary two-tailed one-way ANOVA with Tukey's multiple comparison test; E, ordinary two-tailed two-way ANOVA with Tukey's multiple comparison test). Scale bars: 10 µm.

In order to examine the positions of midbodies in PRL-3-overexpressing MDCK cysts, we examined different midbody markers. When the lumen had not yet opened, the midbody markers MKLP1 (KIF23), Cep55, Aurora kinase B and γ-tubulin marked the central lumen in parental and GFP–PRL-3-C104S MDCK cysts (Fig. 3A,B,D; Fig. S3A,B,E,F), and these proteins were also identified in the ectopic lumens formed in GFP-PRL-3-WT MDCK cysts (Fig. 3C,D; Fig. S3C,G,H). Because Cep55 can be also found in other subcellular structures – e.g. in the centrosome (Martinez-Garay et al., 2006) – and because the antibody against MKLP-1 generates background in immunofluorescence analyses, the colocalization between Cep55 and MKLP1 was quantified in punctate structures in the lumens to ensure that the luminal structures were midbodies (Fig. S3D). Moreover, in MDCK and Caco-2 cysts that overexpressed GFP–PRL-3-WT, ∼30–50% of midbody remnants were incorrectly localized in the lateral membrane compared to the central (apical) localization in parental and GFP–PRL-3-C104S MDCK cysts at early cystogenesis stages (Fig. 3E; Fig. S3I). To follow the position of post-mitotic midbodies over time in morphologically normal PRL-3-C104S and also PRL-3-WT cysts, we next performed live-cell imaging experiments using YFP–MKLP1 as a midbody remnant marker. At the two-cell stage, PRL-3-C104S cysts maintained the post-mitotic midbody at their center (Fig. 3F, arrow; see also Movie 2). In contrast, PRL-3-WT overexpression altered the post-mitotic midbody position from the apical to the lateral membrane, with subsequent formation of PRL-3-rich patches, previously identified as AMISs, around the midbody at that position (Fig. 3G, arrow; see also Movie 3). These results corroborate our observation that the ectopic lumens arise at the lateral membrane (Fig. 2E).

Interestingly, in Fig. 3F, the first midbody (arrow) was maintained over time at the edge of the lumen after subsequent divisions. Therefore, we examined midbody markers at a later cystogenesis stage in MDCK cysts. We observed that midbody remnants persisted after lumen opening, delineating the edge of the central and also ectopic lumens in parental and PRL-3-overexpressing cysts (Fig. 3H–K; Fig. S3J–M, see also Movie 4), similar to previous observations in proliferative zones of epithelial surfaces in mice (Maliga et al., 2013). This could suggest that in addition to positioning the apical membrane, midbodies could have a role in lumen and cell polarity maintenance given that they persist at the edge of the lumen instead of being internalized or degraded.

Taken together, these results show that PRL-3 activity leads to lateral mispositioning of the midbody, resulting in ectopic lumen formation.

### PRL-3 alters the post-mitotic midbody position by accelerating cytokinesis timing

**Fig. 4.**
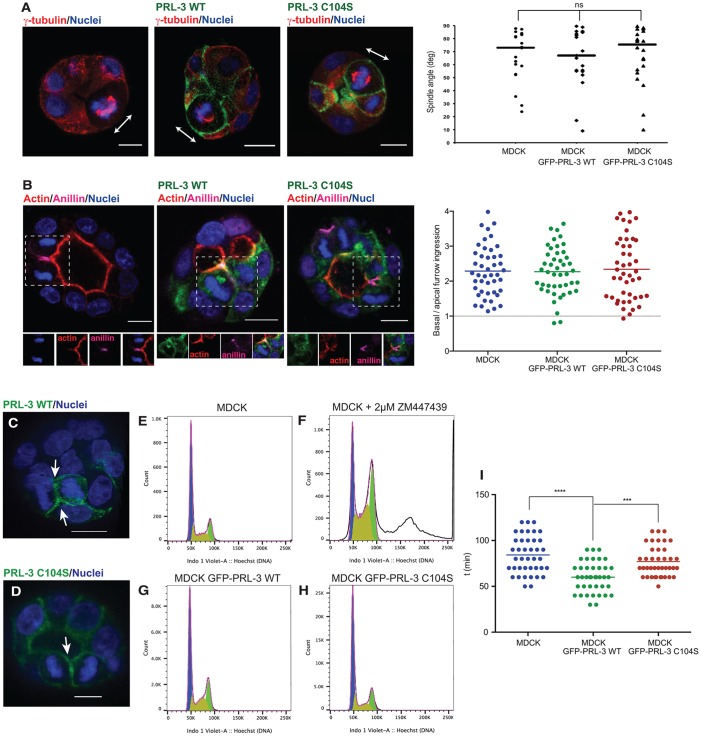
**PRL-3 accelerates cytokinesis.** (A) Representative images of metaphase cells in 72-h parental MDCK cysts or cysts that stably expressed GFP–PRL-3-WT or GFP–PRL-3-C104S (green). Blue, nuclei; red, γ-tubulin. Arrows indicate the orientation of the mitotic spindles. The spindle angle was analyzed. deg, degree. MDCK and PRL-3-C104S, *n*=21; PRL-3-WT, *n*=22. Scale bars: 10 µm. (B) Representative images of cells undergoing cytokinesis in 72-h parental MDCK cysts or cysts that stably expressed GFP–PRL-3-WT or GFP–PRL-3-C104S (green). Blue, nuclei; red, actin; magenta, anillin (cleavage furrow). Dashed boxes highlight the cell undergoing abscission. Merged and split channels of the selected region are shown below the picture. Asymmetry was measured when the cleavage furrow was around 50% ingressed (measured as distance between apical and basal membrane). A line tangent to the apical membrane and another tangent to the basal membrane were drawn. The distance between each line and the cleavage furrow was measured to determine the ingression for both the basal and the apical side, and the ratio between the basal and apical membrane ingression was plotted. Symmetric abscission=1. *n*=45. Scale bars: 10 µm. (C,D) 48-h MDCK cysts overexpressing GFP–PRL-3-WT (C) or GFP–PRL-3-C104S (D). Arrows indicate PRL-3 accumulation in the ingressed furrow. *n*=30. See also Movie 5. Scale bars: 10 µm. (E–H) Cell cycle analysis. MDCK cells (E), MDCK cells treated with 2 μM of ZM447439 (Aurora kinase B inhibitor) over 48 h (F) and MDCK cells that stably expressed GFP–PRL-3-WT (G) or GFP–PRL-3-C104S (H) were analyzed by performing flow cytometry. Blue, G0 and/or G1; olive green, S phase; green, G2; white, tetraploid cell. (I) The time taken for cells to undergo abscission was measured in MDCK cysts and MDCK cysts expressing GFP–PRL-3-WT or GFP–PRL-3-C104S that had also been transfected with Aurora kinase B to track cytokinesis over time. See also Movies 6 and 7. *n*=50. Median (A) or mean (B,I) values for each condition from three biological replicates is shown as a line. ns, not significant; ***P≤0.001; ****P≤0.0001 (A,B,I, ordinary two-tailed one-way ANOVA with Tukey's multiple comparison test).

Planar orientation of the mitotic spindle during metaphase and asymmetric cell division is required to localize the midbody at the apical membrane (Bryant et al., 2010; Jaffe et al., 2008; Li et al., 2014; Overeem et al., 2015; Schlüter et al., 2009). Mitotic spindle misorientation has been extensively studied because it is implicated in epithelial tumorigenesis (Noatynska et al., 2012; Pease and Tirnauer, 2011). Indeed, knockdown of proteins like Par3 or Cdc42 impairs spindle orientation, showing a similar phenotype (Hao et al., 2010; Jaffe et al., 2008) to that which we observe with PRL-3 overexpression. Thus, we determined whether aberrant expression of GFP–PRL-3-WT leads to the misorientation of the mitotic spindle. In GFP–PRL-3-WT-overexpressing MDCK cysts, mitotic spindles that were measured at metaphase as previously described (Hao et al., 2010; Jaffe et al., 2008; Rodriguez-Fraticelli et al., 2010; Wei et al., 2012; Zheng et al., 2010) were predominantly perpendicular to the apical–basal axis (Fig. 4A), suggesting that PRL-3 might lead to the failure of the apical delivery of the midbody by altering the asymmetric abscission, a process that so far has been a theoretical scenario (Jaffe et al., 2008; Overeem et al., 2015). Accordingly, furrow ingression was measured in MDCK cysts, as previously described (Liu et al., 2012), but GFP–PRL-3-WT did not result in a shift of asymmetric abscission when overexpressed, compared to parental and GFP–PRL-3-C104S-overexpressing MDCK cysts (Fig. 4B). Thus, PRL-3 must alter midbody positioning through an unknown alternative mechanism.

We noted that during cytokinesis, PRL-3-WT and PRL-3-C104S were always (*n*=30) recruited to the cytokinetic bridge (Fig. 4C,D; see also Movie 5). Therefore, we studied whether PRL-3 overexpression affects the cytokinesis process. An effect of PRL-3 in cytokinesis has never been addressed previously. At early cytokinetic stages, Aurora kinase B is activated by phosphorylation. It in turn phosphorylates MKLP1, preventing cleavage furrow regression (Steigemann et al., 2009). ZM447439, an Aurora kinase B inhibitor, prevents MKLP1 phosphorylation, leading to tetraploidization as a result of furrow regression (Ikezoe et al., 2007; Steigemann et al., 2009). Accordingly, in order to study whether PRL-3 prevents abscission, the tetraploid MDCK cell population (2D culture) was measured by performing flow cytometry analysis. As expected, the tetraploid cell population increased in ZM447439-treated compared to the non-treated MDCK cells (Fig. 4E,F). When GFP–PRL-3 WT was overexpressed, the population of tetraploid cells was comparable to that in non-treated parental and GFP–PRL-3-C104S MDCK cells (Fig. 4E–H). Thus, we did not observe prevention of abscission by PRL-3. We then measured the abscission time in 2D-cultured parental MDCK cells, as well as in GFP–PRL-3-WT- and GFP–PRL-3-C104S-overexpressing cells. Fig. 4I shows that abscission was faster when PRL-3-WT was overexpressed (see also Movies 6 and 7). This could in turn result in a new scenario in which abscission takes place earlier than the apical positioning of the midbody, leading to lateral ectopic lumen formation.

## DISCUSSION

PRL-3 protein is either not expressed or expressed at low levels in healthy human epithelial tissue (Matter et al., 2001; Rios et al., 2013). Furthermore, in MDCK and Caco-2 epithelial cell lines, which have been extensively used for cell polarity studies, PRL-3 was undetectable at the protein level, and knockdown did not show any effect, showing that PRL-3 is not required for the establishment of cell polarity. However, PRL-3 is found overexpressed in many epithelial cancers (Al-Aidaroos and Zeng, 2010; Rios et al., 2013). Here, we found that the breast cancer cell line MCF-7 that expresses detectable levels of PRL-3 protein (Geiger et al., 2012; Wang et al., 2010) shows multiple-lumen-containing branch ends when grown in Matrigel. This phenotype can be rescued by inducible knockdown of PRL-3 and with the PRL inhibitor analog 3, suggesting that aberrant PRL-3 expression disrupts the epithelial architecture. In addition, the strong phenotype when overexpressing PRL-3 in the non-cancerous MDCK cysts suggests that PRL-3 expression in healthy epithelial tissue is detrimental and has to be avoided, explaining its absence in healthy epithelial tissue. Whether PRL-3 is a bona fide oncogene has not yet been addressed in detail, although a report using PRL-3-knockout mice shows that the absence of the PRL-3 gene leads to a 50% decrease of tumor formation in a colon cancer model (Zimmerman et al., 2013). Our data support these findings given that the overexpression of PRL-3 in the non-cancerous background (MDCK cysts) causes invasiveness and detrimental disorganization of epithelial structures, which are both fundamental events in tumor progression (McCaffrey and Macara, 2011).

In order to answer how PRL-3 activity causes the disruption of epithelial architecture, knowledge of its cellular substrates is required. The identification of PRL-3 substrates has remained extremely challenging since its discovery over a decade ago (Al-Aidaroos and Zeng, 2010; Rios et al., 2013). In the context of lumen formation, the suggested PRL-3 substrates PI(4,5)P_2_ and ezrin are known determinants of cellular polarity (Martin-Belmonte et al., 2007). Nevertheless, we did not observe substantial changes in the phosphorylation status of PI(4,5)P_2_ and ezrin when overexpressing PRL-3. Rapid changes in tightly controlled phosphorylation levels, as during cleavage furrow ingression during asymmetric abscission, could, however, be difficult to detect, and it will take the development of elaborate and sensitive methods to make progress in this area.

**Fig. 5.**
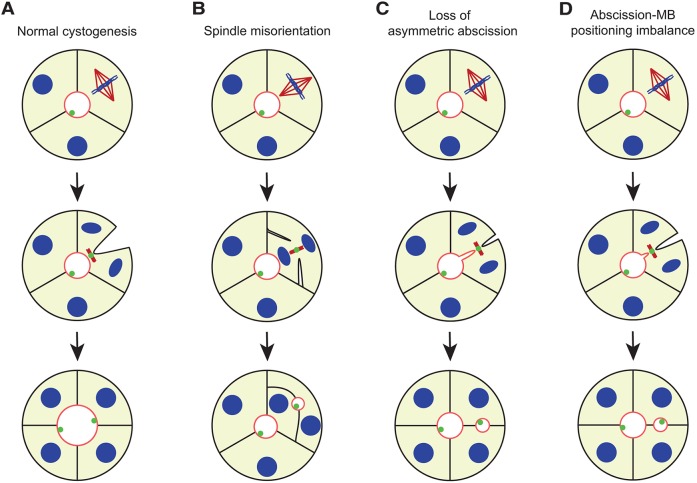
**Mechanisms for ectopic lumen formation through post-mitotic midbody mispositioning.** During normal cystogenesis, the mitotic spindle is positioned perpendicular to the apical–basal axis, followed by asymmetric furrow ingression for apical delivery of the midbody to ensure maintenance of a single lumen (A). Loss of mitotic spindle orientation (B) or loss of asymmetric furrow ingression (C) can lead to failure in apical midbody positioning and an ectopic lumen is formed. Moreover, we propose a new mechanism in which cytokinesis occurs earlier, and abscission takes place before the midbody reaches its luminal position (D). Midbodies (MBs) from previous cell divisions remain at the apical surface. Apical membrane, red; γ-tubulin, dark red; nuclei and chromosomes, blue; midbody and midbody remnants, green. Adapted from Jaffe et al. (2008). This figure is published under a Creative Commons BY-NC-SA license (https://creativecommons.org/licenses/by-nc-sa/3.0/).

During cell division of polarized cells, endosomes containing apical components move along central spindle microtubules to fuse with the cleavage furrow plasma membrane around the midbody, which would finally generate an apical lumen (Li et al., 2014). In further cell divisions, positioning of the midbody in the apical membrane is required for correct lumenogenesis and has been proposed to be achieved through the combination of planar mitotic spindle orientation and asymmetric cleavage furrow ingression (Fig. 5A) (Bryant et al., 2010; Jaffe et al., 2008; Morais-de-Sá and Sunkel, 2013). Polarity proteins such as Par3 and Cdc42 have been reported to be important for establishment of correct spindle orientation (Fig. 5B), whereas spindle-independent midbody mispositioning remains a theoretical model (Fig. 5C) (Dionne et al., 2015; Hao et al., 2010; Jaffe et al., 2008). However, PRL-3 overexpression disrupted neither spindle orientation nor asymmetric cleavage furrow ingression, suggesting that a third alternative mechanism for midbody mispositioning might exist. Cytokinesis plays a key role in midbody apical positioning (Li et al., 2014; Overeem et al., 2015; Wang et al., 2014). Here, we show that PRL-3 localizes to the cytokinetic bridge and accelerates abscission timing. Based on these findings, we propose that prevention of the apical positioning of the midbody and completion of abscission in the lateral membrane could be caused by faster cytokinesis (Fig. 5D).

The molecular mechanisms underlying the timing of asymmetric abscission in epithelial cells are thought to be complex and include an abscission checkpoint that is still not well understood (Caballe et al., 2015; Lafaurie-Janvore et al., 2013). We did not observe a defect in the ploidy of the cells, which makes a disruption of the furrow regression rather unlikely. One possible scenario is that PRL-3 could be acting downstream of Aurora kinase B, causing a failure in abscission delay – for example, by counteracting Unc-51-like kinase 3, which is needed for the final stages of abscission (Caballe et al., 2015). In general, the knowledge of the roles and involvement of particular phosphatases in cytokinesis is very limited (Caballe et al., 2015). However, although PRL-3 localizes to the site of cytokinesis, it is unclear if its involvement is direct or indirect given that PRL-3 expression in cells affects a plethora of pathways (Rios et al., 2013). This new direction in PRL-3 research will be addressed in future studies.

For the first time, we provide experimental evidence for an alternative model for the disruption of cystogenesis by ruling out the previously reported models and showing that the overexpression of the cancer-promoting phosphatase PRL-3 leads to faster abscission. Moreover, it has been suggested previously that midbody remnants can participate in non-cytokinetic functions, such as cell fate determination and cell polarity specification (Chen et al., 2013; Crowell et al., 2014; Kuo et al., 2011; Morais-de-Sá and Sunkel, 2013). In line with this, our results confirm that midbodies have essential functions in *de novo* lumenogenesis and lumen growth during cytokinesis, and right after abscission (Jaffe et al., 2008; Li et al., 2014; Schlüter et al., 2009), and suggest that they play a role long after cell division in polarity maintenance or specification given that, instead of being degraded, the midbody remnants delineate the apical membrane.

In conclusion, we identify the first enzyme, PRL-3, that is involved in epithelial architecture disruption through midbody mislocalization that does so without perturbing the mitotic spindle orientation or asymmetric cleavage furrow ingression. Instead, we suggest a new alternative scenario in which abscission takes place before the midbody can reach the apical position, leading to lateral retention of the midbody. Disruption of epithelial architecture by PRL-3 is a newly recognized mechanism through which PRL-3 can contribute to cancer progression.

## MATERIALS AND METHODS

### Cell culture

MDCK cells were grown in minimum essential medium (MEM) containing 10% fetal bovine serum (FBS) (both from Gibco, Life Technologies), 100 U/ml penicillin and 100 μg/ml streptomycin (Sigma) and 2 mM L-glutamine (Sigma). The Caco-2 cell line was cultured in Dulbecco's modified Eagle's medium (DMEM) containing 4.5 g/l glucose, 10% FBS, 100 U/ml penicillin and 100 μg/ml streptomycin, 2 mM L-glutamine and 0.01 mg/ml transferrin (BD Bioscience). The MCF-7 cell line was cultured in DMEM containing 4.5 g/l glucose, 10% FBS, 100 U/ml penicillin and 100 μg/ml streptomycin and 2 mM L-glutamine. MDCK cells were provided by Fernando Martín-Belmonte [Centro de Biología Molecular Severo Ochoa, Consejo Superior de Investigaciones Científicas (CSIC), Madrid, Spain] and Caco-2 and MCF-7 cells were available at EMBL Heidelberg. MDCK and Caco-2 cells that stably expressed GFP–PRL-3 (either WT or C104S) were generated through transfection (with Lipofectamine LTX, Life Technologies) of pEGFP-C1-PRL-3-WT or pEGFP-C1-PRL-3-C104S accordingly. MDCK cells that stably co-expressed GFP–PH-PLCδ and mKate–PRL-3 (either WT or C104S) were generated through transfection of pEGFP-PH-PLCδ with pmKATE2-C-PRL-3 (WT or C104S) accordingly, and double selection with 4 mg/ml of Geneticin and 5 μg/ml of Blasticidin S (Invitrogen) for 2 weeks. In all cases, resistant clones were selected with 4 mg/ml of Geneticin (Gibco, Life Technologies) for 2 weeks, and a pool of GFP-positive cells was isolated by using fluorescence-activated cell sorting. All cell lines were regularly authenticated by examining the formation of the characteristic morphology in 3D culture and were tested for mycoplasma contamination (Lonza MycoAlert; catalog no. LT07-118).

### Cyst Matrigel culture

Cell cyst formation was performed as described previously (Martin-Belmonte et al., 2007). Briefly, MDCK, Caco-2 or MCF-7 cells were trypsinized to a single cell suspension of 5×10^4^ cells/ml in complete medium containing 2% for MDCK, 5% for Caco-2 or 10% for MCF-7 of Matrigel (BD Bioscience). Suspensions (250 μl) were plated onto 8-well coverglass chambers (Nunc), pre-coated with 10% Matrigel in MEM serum-free for MDCK and Caco-2 cells, or with 100% Matrigel for MCF-7 cells. In order to inhibit PRL-3, analog 3 (Enamine) was added to MDCK, Caco-2 and MCF-7 cysts.

### Plasmids and PRL-3 knockdown

Vectors pEGFP-C1 and pmKATE2-C were used to overexpress WT human PRL-3. The PRL-3-C104S mutant was generated by using site-directed mutagenesis (McParland et al., 2011). YFP–MKLP1 was a gift from Dr Rainer Pepperkok (EMBL, Heidelberg, Germany). For transient pmKATE2-C-PRL-3-WT and YFP–MKLP1 expression, MDCK cells in 2D culture were transfected (Lipofectamine LTX, Life Technologies) before plating for 3D culture. For transient pEGFP-PRL-1-WT and pEGFP-PRL-3-WT expression, MDCK cells in 2D were transfected by electroporation using Amaxa SF Cell Line 4D-Nucleofector kit in a 4D-Nucleofector Core Unit instrument and the transfection program CA-152 (Lonza) before plating for 3D culture. Synthetic double-stranded small interfering (si)RNA was commercially obtained from Ambion (Life Technologies): Silencer Select Negative Control #1 siRNA and PRL-3 siRNA (sense 5′-CCUUCAUUGAGGACCUGAATT-3′). Both siRNAs were used at 10 nM. In MDCK cells, siRNAs were transfected by electroporating cells using the Amaxa SF Cell Line 4D-Nucleofector kit in a 4D-Nucleofector Core Unit instrument and the transfection program CA-152 (Lonza). In Caco-2 cells, siRNAs were transfected with jetPRIME (Polyplus transfection). The PRL-3 mRNA level was determined by performing one-step reverse transcription (RT)-PCR (Qiagen). For inducible PRL-3 knockdown in MCF-7 cells, stable expression of small hairpin (sh)RNA against PRL-3 was established. MISSION 3X LacO Inducible Non-Target shRNA Control plasmid DNA (Sigma, catalog no. SHC332) was used as a control shRNA and, for PRL-3 knockdown shRNA, sense sequence 5′-CCGGAGCTCACCTACCTGGAGAAATCTCGAGATTTCTCCAGGTAGGTGAGCTTTTTTG-3′, extracted from MISSION shRNA plasmid DNA, was inserted into the isopropyl β-D-1-thiogalactopyranoside (IPTG)-inducible vector pLKO-puro-IPTG-3×LacO. MCF-7 cells were transfected (Lipofectamine LTX, Life Technologies) with both constructs, resistant clones were selected with 1 μg/ml of puromycin dihydrochloride (Santa Cruz Biotechnology), and single clones were expanded and selected by performing OneStep RT-PCR analysis (Qiagen) after 4 days of induction with 1 mM IPTG (Peqlab). IPTG-containing medium was replaced with fresh every 2 days.

### Antibodies and immunolabeling

Primary mouse antibodies used were: anti-Sec8 (1:100; Enzo Life Sciences; catalog no. ADI-VAM-SV016; clone 14G1; lot 08010910), which also nonspecifically stained nuclear structures; anti-Aurora-kinase-B (1:200; BD Transduction Laboratories; catalog no. 611082; clone 6/AIM-1); anti-γ-tubulin (1:100; Sigma; clone GTU-88) and anti-ezrin (1:100; BD Transduction Bioscience; catalog no. 610602; lot 87352) antibodies. Primary rabbit antibodies used were: anti-MKLP1 (1:1000; Santa Cruz Biotechnology; catalog no. sc-867; clone N-19; lot I1614); anti-α-tubulin (1:500; Millipore; catalog no. 04-1117; clone EP1332Y) and anti-ZO-1 (1:200; Invitrogen; catalog no. 40-2200; clone TJP1) antibodies. An anti-phosphorylated-ERM antibody, which detects phosphorylation of ezrin (at Thr-567), radixin (at Thr-564) and moesin (at Thr-558) (1:100; Abcam; catalog no. ab76247; clone EP2122Y; lot GR14705-13), was used in the experiments to detect phosphorylated ezrin given that MDCK cells express mainly ezrin in terms of ERM-family proteins (Wakayama et al., 2011). Primary goat antibodies used were against Cep55 (1:100; Santa Cruz Biotechnology; catalog no. sc-162675; clone E-14; lot C0813) and anillin (1:25; Abcam; catalog no. ab5910; lot GR270489-1). The detection of PRL-3 using antibodies is known to yield a quite weak signal, and high PRL-3 concentrations are required (Zimmerman et al., 2013). For PRL-3 detection, antibodies used were from Santa Cruz Biotechnologies (1:200; mouse antibody; catalog no. sc-130355; clone 318; lot F2111) and Sigma (1:1000; rabbit antibody; catalog no. P0498; lot 104K4792; and 1:1000; rabbit antibody; catalog no. SAB2101908; lot QC16627), but we did not observe a signal in western blot analyses (data not shown). Alexa-fluorophore-conjugated secondary antibodies (1:1000 for all secondary antibodies; Invitrogen), Rhodamine-conjugated phalloidin (1:1000; Invitrogen; catalog no. R415; lot 1090018) and Hoechst 33342 to label nuclei (10 μg/ml) were also used. Antibody validation details and citations are provided by the manufacturer. The procedure for the immunofluorescence staining of cysts has been previously described in detail (Elia and Lippincott-Schwartz, 2009). Briefly, samples were rinsed with phosphate-buffered saline (PBS) twice and fixed with 4% paraformaldehyde for 30 min. Cells were quenched with 0.1 M glycine in PBS for 20 min, permeabilized with 0.5% Triton X-100 in PBS for 10 min and blocked with 10% FBS in PBS with 0.5% Triton X-100 for 60 min. Samples were then incubated with primary antibodies in blocking solution at 4°C overnight, followed by washing and incubation with Alexa-fluorophore-conjugated secondary antibody for 1 h. Images of the cells were acquired at room temperature (immunofluorescence) on a spinning disc confocal microscope (PerkinElmer Ultraview ERS) using a Plan-Apochromat 63×1.4 NA oil objective. Live-cell images were recorded on the same microscope with a stable monitored temperature of 37°C under 5% CO_2_, acquiring a single plane every 3 min for a total of 90 min in 3D-cultured MDCK cells expressing YFP–MKLP-1 pmKATE-PRL-3-WT and every 5 min for a total of 14 h in 3D-cultured MDCK cells expressing YFP–MKLP-1 pmKATE-PRL-3-C104S. Images were processed using Imaris (Oxford Instruments), Volocity (PerkinElmer) or FIJI software (National Institutes of Health). Contrast or brightness adjustment was applied to the whole image. In FIJI software, a 50-pixel rolling ball radius was used to subtract background and a 1-pixel-radius median filter was applied.

### Measurement of mitotic spindle angle

Mitotic spindle orientation was measured as described previously (Hao et al., 2010; Jaffe et al., 2008; Rodriguez-Fraticelli et al., 2010; Wei et al., 2012; Zheng et al., 2010). Briefly, MDCK cysts were grown and fixed at 72 h, as described for immunolabeling. Anti-γ-tubulin antibody and Hoechst 33342 (nuclei) were used to stain the mitotic spindle. Confocal images of metaphase cells in the middle of the cyst were taken. A line in the direction of the mitotic spindle was drawn using ImageJ. Another radial lane between the center of the cyst and the basal membrane was drawn crossing the first line. The small angle formed between both lines was measured.

### Measurement of cleavage furrow ingression symmetry

Cleavage furrow positioning during cell division was measured as described previously (Liu et al., 2012). Briefly, MDCK cysts were grown and fixed at 72 h, as described for immunolabeling. Anti-anillin antibody (cleavage furrow), Rhodamine–phalloidin (actin) and Hoechst 33342 (nuclei) were used. Confocal images of cells at cytokinesis were taken when the cleavage furrow was around 50% ingressed. A line tangent to the apical membrane and another to the basal membrane were drawn using ImageJ. The distance between each line and the cleavage furrow was measured to obtain apical and basal ingression, and the ratio between the basal and apical membrane ingression was plotted. A value of 1 represents symmetric abscission.

### Measurement of abscission timing

Cytokinesis duration was measured in 2D-cultured non-polarized MDCK cells. mCherry–Aurora-kinase-B was transfected into cells (jetPRIME, Polyplus transfection) to stain the cleavage furrow. Pictures were taken every 10 min during 24 h in an Olympus-ScanRcube5 microscope using a Plan-Apochromat 20×0.7 NA objective with a stable monitored temperature of 37°C under 5% CO_2_. The time between cytokinetic bridge formation and cleavage was measured.

### Immunoblotting

Cells were lysed on ice in HNTG buffer (50 mM Hepes, pH 7.5, 150 mM NaCl, 10% glycerol, 5 mM EGTA and 1% Triton X-100) containing complete protease inhibitors (Roche). Equal amounts of lysate protein were separated by performing SDS-PAGE. Western blotting was performed with different antibodies against PRL-3 and with secondary anti-mouse or anti-rabbit antibodies conjugated to horseradish peroxidase (Sigma and Amersham Biosciences, respectively). Visualization was achieved with the Western Lightning Plus-ECL solution (Perkin Elmer).

### Flow cytometry

To measure the heterogeneous GFP–PRL-3-WT and GFP–PRL-3-C104S protein levels in MDCK cell lines that stably expressed these constructs, flow cytometry was performed. A 3×10^6^-cell sample was resuspended in 200 μl of ice-cold PBS. The GFP content of 2×10^5^ cells was measured by performing flow cytometry (LSR-Fortessa bench top analyzer, BD Bioscience), and live cells were gated using 1 μg/ml 4′,6-Diamidine-2′-phenylindole (DAPI) (Roche, catalog no. 10236276001). Cell cycle analysis was performed as described previously (Schmid and Sakamoto, 2001). Briefly, MDCK cells were resuspended in MEM that lacked Phenol Red but contained 10% fetal bovine serum (FBS) (both from Gibco, Life Technologies), 100 U/ml penicillin and 100 μg/ml streptomycin (Sigma), and 2 mM L-glutamine (Sigma) to a final concentration of 2.6×10^6^ cells/ml. 30 μg/μl of Hoechst 33342 (Sigma) was added to the suspended cell and incubated at 37°C for 45 min. Finally, DNA content was measured by performing flow cytometry (LSR-Fortessa bench top analyzer, BD Bioscience). In order to inhibit Aurora kinase B, MDCK cells were treated with 2 μM ZM447439 (Santa Cruz Biotechnologies) for 48 h before being resuspended in PBS.

### Ca^2+^-switch assay

Ca^2+^-switch experiments were performed as described previously (Chen and Macara, 2005). Briefly, MDCK cells were plated in 8-well Lab-Tek II chambers with complete growth medium (normal Ca^2+^-containing medium; HCM). After 24 h cells, were switched into low-Ca^2+^ medium (LCM). LCM was prepared by supplementing MEM with 2% FBS, which had been dialysed against 150 mM NaCl overnight. After 16 h of incubation in LCM, cells were switched back to HCM for the indicated times.

### MS-based phosphoinositide detection

Phosphoinositide quantification was performed as described previously (Haag et al., 2012). Briefly, MDCK cells were plated in 6-well plates and grown for 5 days in Matrigel. Cells from two 6-well plates were combined and subjected to an acidic–neutral extraction of trichloroacetic acid (TCA)-washed cell pellets using PI(4)P 17:0–20:4 and PI(4,5)P_2_ 17:0–20:4 from Avanti Polar Lipids as internal standards for quantification. Mass spectrometry was performed on a QTRAP 5500 instrument (AB Sciex) equipped with a Triversa NanoMate system (Advion Biosciences). Phosphoinositides were measured by scanning for neutral losses of *m*/*z* 357 (phosphatidylinositol) and *m*/*z* 437 (PIP_2_) on an AB Sciex QTRAP 5500 instrument at collision energies of 25 eV and 35 eV, respectively. Mass spectra were evaluated using LipidView software (AB Sciex). PIP and PIP_2_ amounts were normalized to total phospholipids, which was determined in neutral and acidic phases, as described previously (Özbalci et al., 2013).

### MS-based PRL-detection in MDCK cells

#### Cell lysis, protein digestion and peptide labeling

Three million cells were suspended in lysis buffer (HEPES 200 mM; pH 7.8) containing 1 mg/ml Rapigest SF surfactant (Waters) and a protease inhibitor cocktail (Sigma, P8340). The cells were pipetted through a pipette tip 20 times and subsequently heated for 5 min at 95°C, followed by sonication for 20 min. The proteins were reduced with dithiothreitol, and cysteine residues were carbamidomethylated with iodoacetamide. The reduced and carbamidomethylated proteins were digested with lysyl endopeptidase (Lys-C) (Wako) for 4 h at 37°C followed by a tryptic (Promega) digestion overnight at 37°C. The resulting peptides were desalted and dimethyl-labeled on Sep-Pak tC18 1cc 100 mg cartridges (Waters), as described previously (Boersema et al., 2009). Briefly, the cartridges were washed with 0.6% acetic acid in 80% acetonitrile and equilibrated with 0.6% acetic acid. Afterwards, the peptides were loaded onto the cartridges and washed with 0.6% acetic acid. The three samples were light-, intermediate- or heavy-dimethyl-labeled with either 4% formaldehyde (CH_2_O) (Thermo Fisher Scientific), deuterated formaldehyde (CD_2_O) (Sigma) or heavy deuterated formaldehyde (^13^CH_2_O) (Isotec) in 50 mM sodium phosphate buffer (pH 7.5) containing 0.6 M cyanoborohydride (Sigma) or cyanoborodeuterate (Sigma). The peptides were flushed for 10 min with labeling solution, followed by washing with 0.6% acetic acid and elution with 0.6% acetic acid in 80% acetonitrile.

#### Peptide fractionation by HPLC

Before separation, the dimethyl-labeled samples were mixed in a 1:1:1 ratio and concentrated in a vacuum centrifuge. The pH of the sample was increased to above pH 10 with 25% ammonia (Merck), and the volume was made up to 50 μl with water. The whole sample was separated on an Agilent 1260 infinity high-performance liquid chromatography system equipped with a Waters XBridge C18 3.5 μm 1×100 mm reversed-phase column at a flow rate of 75 μl/min with 20 mM ammonium formate at pH 10 and 100% acetonitrile as buffers. 90 fractions were collected and subsequently concentrated under vacuum to remove the majority of the organic solvent. The fractions were desalted and pooled in one step into 18 pooled fractions. 16 of these were pooled by taking one early-, one middle- and one late- eluting fraction, and two were created by mixing a part of the earliest and latest fractions together.

#### Nano-liquid chromatography with tandem mass spectrometry analysis

The 18 fractions were analyzed on a nanoAcquity UPLC system (Waters) directly connected to an LTQ Orbitrap Velos Pro instrument (Thermo Scientific) via a Proxeon nanospray source. The peptides were first trapped on a nanoAcquity Symmetry C18 5 µm 180 µm×20 mm (Waters) trapping column and further separated on a nanoAcquity BEH C18 1.7 µm 75 µm×200 mm (Waters) analytical column. The mobile phases were 0.1% formic acid in water and 0.1% formic acid in acetonitrile (Biosolve). The applied three-step gradient was 120-min long, ranging from 3% to 40% acetonitrile. The flow rate was 300 nl/min. The eluent was directly introduced into the mass spectrometer through a Pico-Tip emitter 360-µm outer diameter×20-µm inner diameter, 10-µm tip (New Objective). The capillary temperature was set to 300°C, and the applied spray voltage was 2.2 kV. The mass range of the full scan MS spectra was 300–1700 *m*/*z*. MS1 spectra were recorded in profile mode in the Orbitrap. The resolution was set to 30,000. Lock mass correction was used for internal calibration using a background ion at *m*/*z* 445.12003. The 15 most abundant parent ions were subjected to fragmentation by using collision-induced dissociation. The normalized collision energy was set to 40. Charge state screening was enabled with only multiply-charged ions being selected for fragmentation.

#### Data analysis

Raw data were processed with proteome discoverer (PD) software version 1.4.0.288 (Thermo Scientific). The MS2 spectra were filtered with a Top N peaks filter set to 6 with a 100-Da mass window. The resulting data were searched against the UniProt dog database (26106 sequences) and against a database comprising the UniProt human database and common contaminants (86945). The search algorithm applied was Mascot version 2.2 (matrix science). The search parameters were two missed cleavages with trypsin as enzyme; a mass tolerance of 20 ppm for MS1 and 0.5 Da for MS2. Carbamidomethylation was chosen as a fixed modification for cysteine residues, and oxidation on methionine and phosphorylation on serine, threonine and tyrosine as variable modifications. A false discovery rate of 1% was calculated by the percolator algorithm that is implemented in PD. Quantification was also performed with PD software with a mass precision set to 2 ppm. The quantification spectra of all peptides of any PRL isoform were manually checked.

### Statistical analysis

Ordinary two-tailed one- or two-way ANOVA and Tukey's multiple comparison tests were performed to study statistical significance: *P≤0.05, **P≤0.01, ***P≤0.001 and ****P≤0.0001. Statistical analysis was performed and graphs were generated with Prism (GraphPad).
